# An In-Situ Reaction Route to Molecular Level Dispersed Bisimide and ZnO Nanorod Hybrids with Efficient Photo-Induced Charge Transfer

**DOI:** 10.1186/s11671-021-03504-3

**Published:** 2021-03-17

**Authors:** Chunzheng Lv, Lirong He, Jiahong Tang, Feng Yang, Chuhong Zhang

**Affiliations:** 1 State Key Laboratory of Polymer Materials Engineering, Polymer Research Institute, Sichuan University, Chengdu, 610065 China; 2 Superconductivity and New Energy R&D Center (SRDC), Key Laboratory of Advanced Technology of Materials (Ministry of Education of China), Southwest Jiaotong University, Chengdu, 610031 China

**Keywords:** Perylene bisimide, ZnO nanorod, Surface photovoltage spectrum, Electric field-induced surface photovoltage spectrum, Interfacial electric field

## Abstract

As an important photoconductive hybrid material, perylene/ZnO has attracted tremendous attention for photovoltaic-related applications, but generally faces a great challenge to design molecular level dispersed perylenes/ZnO nanohybrids due to easy phase separation between perylenes and ZnO nanocrystals. In this work, we reported an in-situ reaction method to prepare molecular level dispersed H-aggregates of perylene bisimide/ZnO nanorod hybrids. Surface photovoltage and electric field-induced surface photovoltage spectrum show that the photovoltage intensities of nanorod hybrids increased dramatically for 100 times compared with that of pristine perylene bisimide. The enhancement of photovoltage intensities resulting from two aspects: (1) the photo-generated electrons transfer from perylene bisimide to ZnO nanorod due to the electric field formed on the interface of perylene bisimide/ZnO; (2) the H-aggregates of perylene bisimide in ZnO nanorod composites, which is beneficial for photo-generated charge separation and transportation. The introduction of ordered self-assembly thiol-functionalized perylene-3,4,9,10-tetracarboxylic diimide (T-PTCDI)/ ZnO nanorod composites induces a significant improvement in incident photo-to-electron conversion efficiency. This work provides a novel mentality to boost photo-induced charge transfer efficiency, which brings new inspiration for the preparation of the highly efficient solar cell.

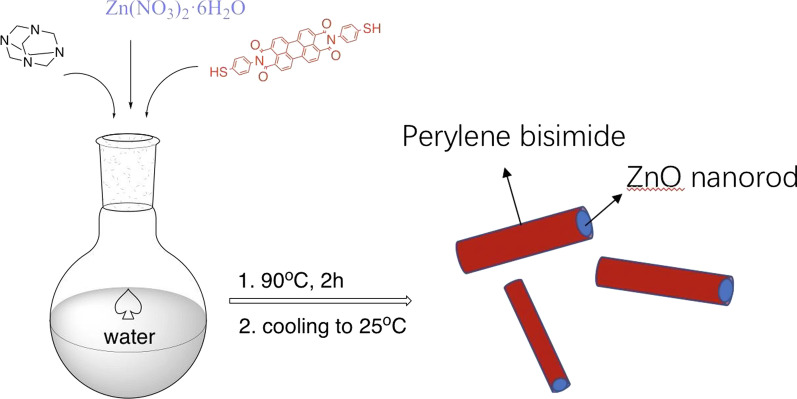

## Introduction

Perylene bisimide is a kind of important functional photovoltaics material which possesses excellent heat stability, chemical and photovoltaics characteristics. Besides, it has a wide spectral absorption range about 450–600 nm and an energy band around 2.5 eV. The lowest unoccupied molecular orbital (LUMO) and the highest unoccupied molecular orbital (HUMO) energy level and large π-conjugated system endow perylene bisimide with high electronic mobility in its stack direction, which may find potential application in organic solar cells [1, 2], field effect transistors [3–5], light-emitting diode [6], self-assembly [7, 8] and bioluminescent probe [9] etc.

Hybridization of organic materials with inorganic nanoparticles usually gives a full play to the best performance of the two entities (e.g., high charge mobility of inorganic semiconductors and excellent light absorption of organic matrixes) in a single hybrid [7, 10, 11]. For instance, inorganic materials tend to process high carrier mobility while organic materials excellent in absorptivity coefficient. By the means of reasonably selecting inorganic materials, composites with coupled and synergistically enhanced function can be fabricated utilizing the unique adsorption and coordination characteristics of organic materials. The valid interface bonding of these kinds of composites makes them possess novel and special properties and usages.

ZnO nanohybrids materials have attracted great attentions in photovoltaic electronics since ZnO nanomaterials have proper energy levels, low costs and an easy preparation process [12, 13]. Among the ZnO-based organic–inorganic hybrids, perylene/ZnO hybrids as photoconductive hybrid materials were intensively studied [14–18] and showed very promising applications as cathode interlayer for high-performance solar cells. However, it is still challenging to obtain highly dispersed (molecular level) perylene/ZnO hybrids due to strong pi-pi stacking of perylene-induced phase separation between perylenes and ZnO [19]. On another hand, molecular level hybridization between perylene and ZnO will be helpful for efficient charge transfer in the hybrids, which has a large impact on photovoltaic property of materials, which is therefore essential to their applications in photovoltaics field [20–22].

**Fig.1:**
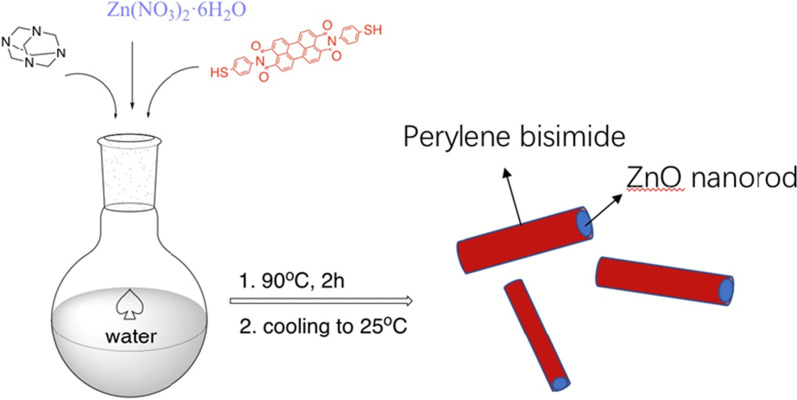
In-situ ZnO nanocrystals growing reaction in the T-PTCD solutions

In this study, we have fabricated molecular level dispersed T-PTCDI/ZnO composite through an in-situ ZnO nanocrystals growing reaction in the T-PTCD solution (Fig. 1). Efficient photo-induced charge transfer between T-PTCDI and ZnO nanorod was observed by means of surface photovoltage, fluorescence spectrum and electric field-induced surface photovoltage spectrum. This study provides a novel and convenient method to prepare molecular level dispersed perylenes/ZnO nanohybrids, which paves a promising way for perylene bisimide/ZnO nanocrystal-based photovoltaics fabrications and applications.

## Methods

### Materials

4-Aminothiophenol (analytically pure), Perylene-3,4,9,10-tetracarboxylic dianhydride (analytically pure), zinc nitrate (analytically pure) and hexamethylenetetramine (analytically pure) were supplied from Aladdin. All the materials were applied directly without further treatment.

### Synthesis of Organic Molecule T-PTCDI

**Fig. 2:**
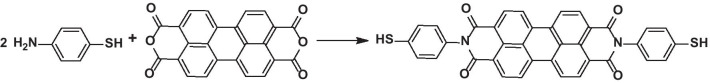
Schematic diagram of the synthetic route for T-PTCDI

The T-PTCDI was synthesized following the steps given by the reference [5], and the molecular structure is shown in Fig. 2.

### Synthesis of T-PTCDI/ZnO Nanorod Composite

Specifically, zinc nitrate (12.5 mmol), hexamethylenetetramine (12.5 mmol) and T-PTCDI (5 mg) were dissolved in a round-bottom flask. Then the mixture was stirred at 90 °C with an agitation rate of 73 rpm. After 2 h, the reaction mixture was cooled to room temperature and remove unreacted salt with water. At last, the product was obtained after been vacuum dried at 50 °C for 24 h. The specific formulation is described in Table 1.

**Table 1:** _Chemical reagents for synthesis of T-PTCDI/ZnO nanorod composite_

Reagents	Dosage	Solvent
Zn(NO_3_)_2_·6H_2_O	0.0125 mol	50 mL water
C_6_H_12_N_4_	0.0125 mol	50 mL water
T-PTCDI	5 mg	–

### Fabrication of Solar Cells

Compacted ZnO was prepared by sputtering on the conductive side of the FTO [23]. An active colloidal dispersion was prepared by adding 10 mL of deionized water to 1.5 g of T-PTCDI/ZnO nanorod composites. The above colloidal dispersion was spread on the surface of compact film by means of the doctor blading technique. The thickness of the obtained porous film was approximately 3 μm. The T-PTCDI/ZnO nanorod composites FTO electrode and platinized counter electrode were assembled into a sealed sandwich-type cell with a gap of a hot-melt ionomer film (Surlyn 1702, thickness 25 mm, DuPont). The electrolyte solution consists a mixture of 0.5 M 2, 3-dimethyl-1-propyl imidazolium iodide, 0.05 M I_2_, 0.1 M LiI in acetonitrile.

### Measurements

The crystalline phases of these samples were characterized by X-ray diffraction (XRD) employing a scanning range from 5° to 75°, and using a MAC Science MXP-3VA diffractometer equipped with a graphite monochromatized CuKα radiation (*λ* = 1.5405 Å) which operated at 40 mA and 40 kV. Further morphology and structural analysis of the products were performed by transmission electron microscopy (TEM) and selected-area electron diffraction (SAED) on JEOL 200CX TEM at an acceleration voltage of 200 kV. UV–visible absorption spectrum was measured by ultraviolet–visible spectrophotometer (Varian CARY 100 Bio). Surface photovoltaic spectra (SPS) were measured on the basis of a lock-in amplifier. The measurement system consists of a sample chamber, a lock-in amplifier (SR830, Stanford Research Systems, Inc.) with a light chopper (SR540, Stanford Research Systems, Inc.) and a source of monochromatic light provided by a 500 W xenon lamp (CHFXM500, Trusttech) and a monochromator (SBP500, Zolix).

## Results and Discussion

**Fig. 3:**
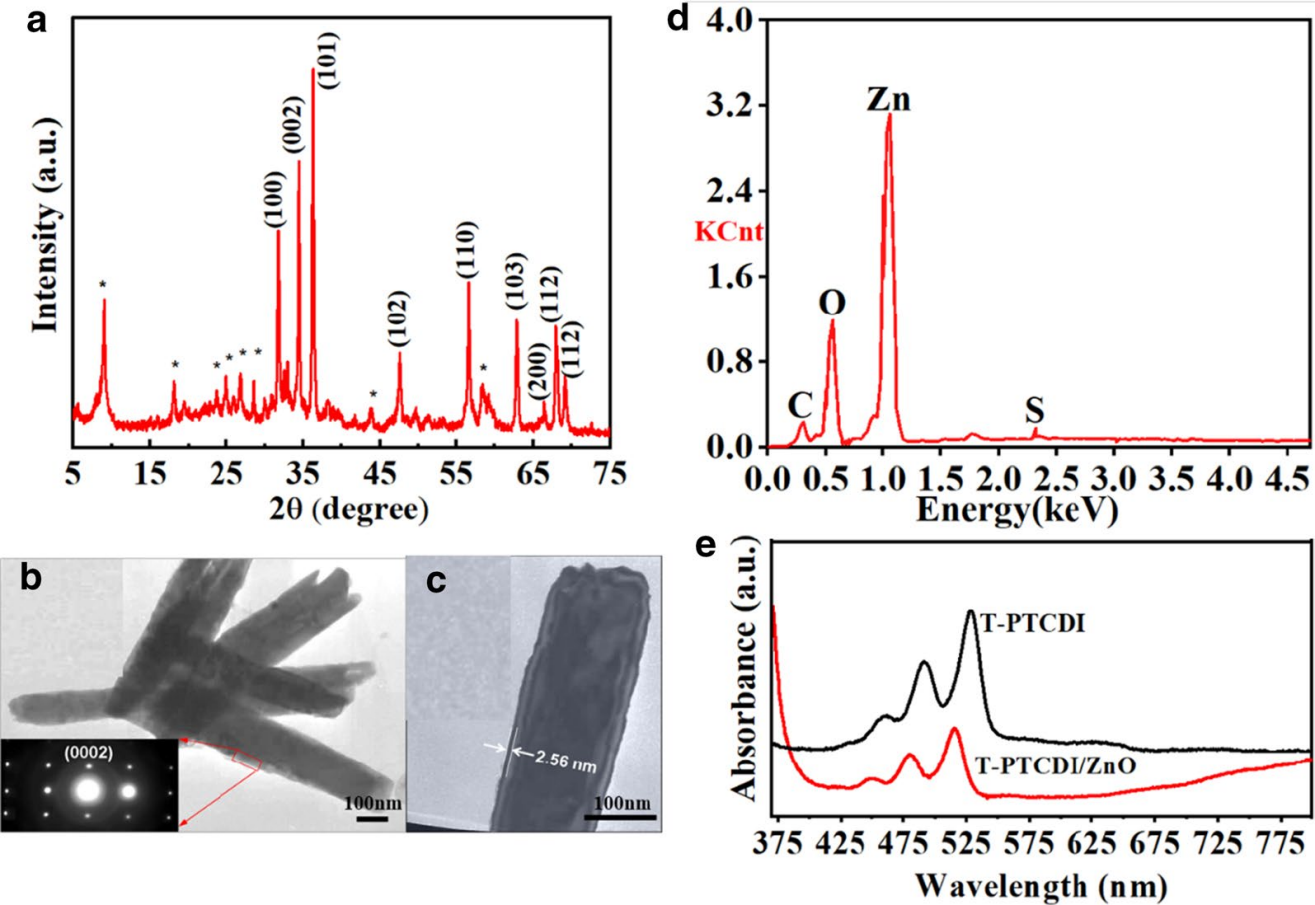
**a** XRD diffraction pattern for ZnO-T-PTCDI composite. TEM of composite material of ZnO-T-PTCDI: **b** low magnification, inset: Selected Area Electron Diffraction Pattern (SAED) and **c** high resolution. **d** Energy spectrum analysis of ZnO-T-PTCDI composite. **e** UV–visible spectra of T-PTCDI and T-PTCDI/ZnO nanorod composites

The resulted sample prepared via in-situ ZnO nanocrystals growing was characterized by XRD, TEM and XPS spectrums as shown in Fig. 3a–d, respectively. Figure 3a shows the XRD diffraction pattern of ZnO-T-PTCDI composite material. It shows the indexed diffraction peak of the composite product and the hexagonal wurtzite structure of ZnO (JCPDS No. 36-1451). In addition to that, there are many diffraction peaks marked with *, which may belong to T-PTCDI. Due to the complicated arrangement of organic molecules, it is difficult to assign these diffraction peaks. Figure 3b, c shows the transmission electron microscopy (TEM) photograph of the ZnO-T-PTCDI composite. Inset picture in Fig. 3b is the Selected Area Electron Diffraction Pattern (SAED). It is determined from SAED diffraction that the growth direction of nanorods is the dominant [0001] direction, and T-PTCDI does not affect the growth of ZnO. It can be seen that the composite material morphology is rod-shaped and covered with a layer of coating material on the surface (Fig. 3c). The TEM images show that the thickness of T-PTCDI is about 2.56 nm (Fig. 3c), which is consistent with the length of T-PTCDI molecules (2.38 nm). Combined with SAED, it is demonstrated that ZnO is nanorod with T-PTCDI molecular film on the outer surface. As revealed in Fig. 3d, electronic energy spectrum analysis (EDAX) result shows the dependence of counting rate of characteristic X-ray photons (KCnt) on energy (kev) for the composite material, demonstrating the containing of C and S elements.

Figure 3e illustrates the UV–visible spectra of T-PTCDI in dilute solution of chloroform. The three Q-Band absorption peaks are 528 nm (Q0,0), 491 nm (Q1,0) and 458 nm (Q2,0) respectively. Perylene bisimide compounds process the flat structure of perylene matrix, in which the π-electrons from mercapto group conjugated with the π-electrons from perylene and form a lager π conjugated system. Therefore, the essence of the electron absorption of perylene bisimide chromophore at visible region is the π–π* transition in the conjugated system.

Compared with the spectra of T-PTCDI in Fig. 3e, it can be seen that all the absorption peak of T-PTCDI/ZnO composite display a blue shift range from 8 to 13 nm and end up locate at 515, 480 and 450 nm, respectively. The blue shift for absorption peak indicates that the agglomeration status of T-PTCDI in the hybridization system has changed in comparison with the pure one and has turned into the H aggregation. The molecular orbital of perylene matrix consists of three HOMO and three LUMO. It has been demonstrated that all the three LUMO are at degenerate energy level and usually exhibit electrons donor feature, whose band gaps are determined to be 2.23 eV according to their absorption edges. The absorption edge commerce at 400 nm in Fig. 3e belongs to the ZnO nanorod.

**Fig. 4:**
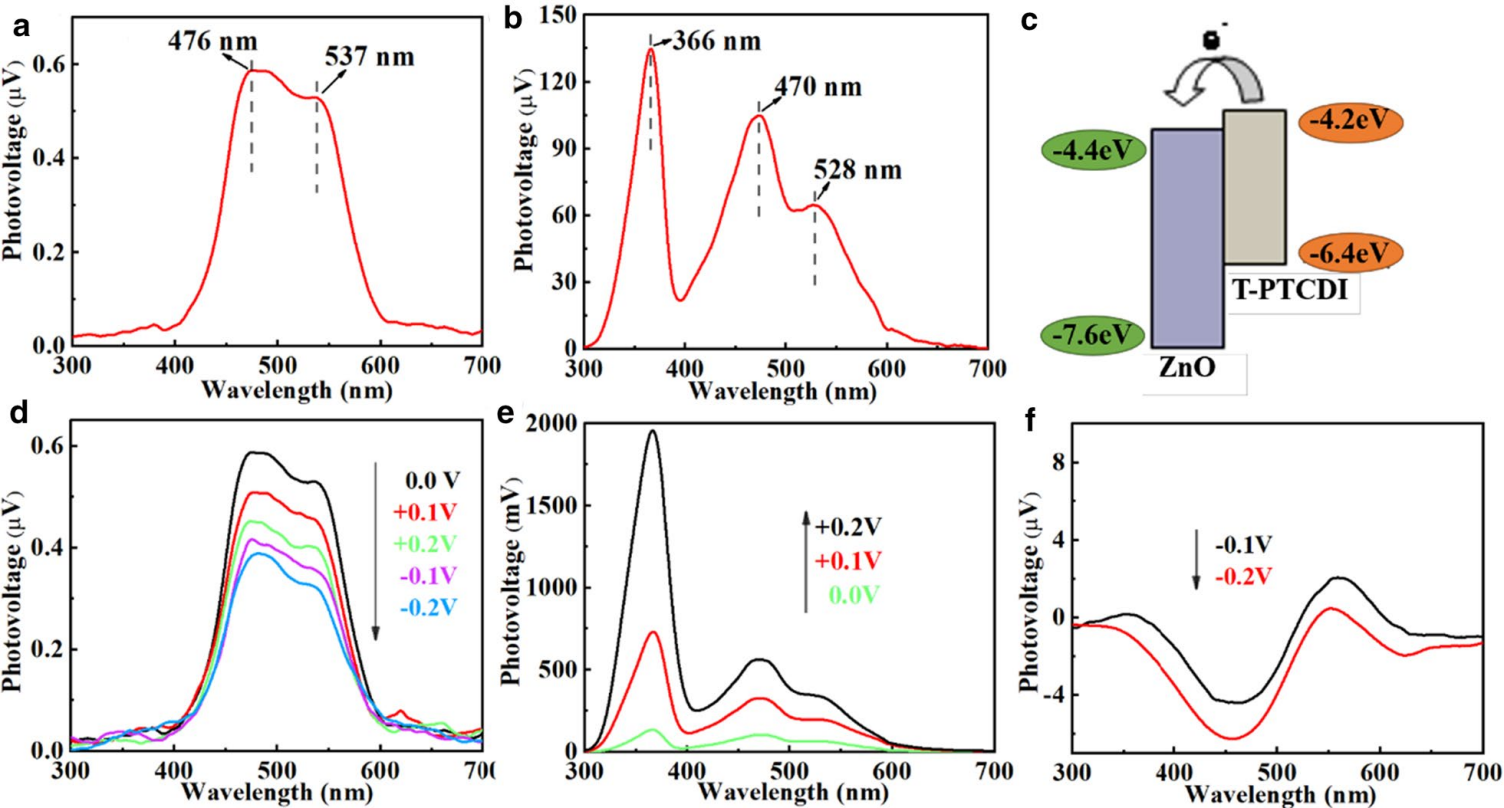
SPS of **a** T-PTCDI and **b** T-PRCDI/ZnO nanorod composites, **c** energy-level alignment of ZnO and T-PTCDI. **d** FISPS of T-PTCDI and **e**, **f** T-PRCDI/ZnO nanorod composites

Figure 4a shows the surface photovoltage spectrum of the T-PTCDI. It is found that the photovoltaic response positions are at 476 nm (Y1) and 537 nm (Y2), respectively, with the response intensity about 0.5–0.6 μV. The locations of absorption peaks are different from that in the UV–visible light absorption spectra, and the response at 476 nm is slightly stronger than that at 537 nm. Figure 4b illustrates the surface photovoltage spectrum of the T-PTCDI/ZnO composite. Obviously, there is a sharp absorption peak at 366 nm, which belongs to the band-to-band transition of the ZnO with the maximum response of 0.13 mV. Compared with the response peak of T-PTCDI, Y2 exhibits a blue shift from 537 to 528 nm while Y1 also changed from 476 to 470 nm with a more pronounced peak profile. Moreover, no obvious change has been observed in the peak position of the ZnO. After comparing the response strength of Y1 and Y2, it can be found that the intensity of Y1 raised from 0.55 to 105 μV after combination, showing a nearly 200 times increase. Also, the response value of Y2 increased from 0.5 to 65 μV, showing an approximately 100 times increase. The built-in potential is closely related to the surface charge density, which can be explained by the formula (1)

1 $$V_{{\text{s}}} = eN_{{\text{s}}}^{2} /2k\varepsilon_{0} \left( {N_{{\text{D}}} - N_{{\text{A}}} } \right)$$

where *V*_s_ is the built-in potential at the Schottky barrier junction, *e* is the electron charge, *k* is the dielectric constant, *ϵ*_0_ is the permittivity of free space, *N*_A_ is the concentration of ionized acceptors, *N*_D_ is the donor concentration and *N*_*S*_ is the density of surface charge. According to Eq. 1, *Vs* enhances with the increase of *N*_S_ since $N_{{\text{D}}} - N_{{\text{A}}}$ is an approximate constant, namely, the surface band bending increases. As a result of that, the separation efficiency of photogenerated carriers has been greatly improved which leads to the effective enhancement of response for SPV [24].

In terms of the difference in the two responsive increases of T-PTCDI, Y1 has a wider band gap due to the higher potential energy barrier formed with ZnO. However, Y2 locates at the edge of LUMO energy level with lower potential energy barrier, which induces the difference in separation effect of electric charges from Y1. According to the principle of surface photovoltaics test, photo-generated electron hole pair will be formed after semiconductors absorb photons. By the effect of built-in field or other electric fields, the electron–hole pair separates and moves in opposite direction, causing variation of the photo-generated potential at the surface. Hence, after ZnO nanorod hybridizes with T-PTCDI, surface photovoltaic charges increase along with the enhancement of the photovoltaic effect, indicating that there exists a high efficiency charge transfer process in the T-PTCDI/ZnO composite.

According to Fig. 4a, the photovoltaic response of the pure T-PTCDI is weak, meaning that the T-PTCDI can only trigger tiny built-in field by itself. It is obvious that the photovoltaics response enhancement of hybridized T-PTCDI is highly possibly driven by the interfacial electrical field formed between the T-PTCDI and ZnO nanorod as well as the agglomeration variation of the T-PTCDI, rather than the built-in field generated by T-PTCDI itself.

Comparing the energy level of ZnO and T-PTCDI, the LUMO energy level of the T-PTCDI is − 4.2 eV, which is higher than the conduction band of ZnO (− 4.4 eV) and therefore an interfacial electric field directed from T-PTCDI to ZnO can be formed between these two entities (Fig. 4c). From another perspective of view, the electron mobility between these two components is varied dramatically. In detail, the electron mobility of the T-PTCDI is under 2.1 cm^2^ V^−1^ s^−1^, while ZnO possesses a high electron mobility range from 200 to 400 cm^2^ V^−1^ s^−1^. When the two entities hybridize with each other, electrons will enrich on the ZnO, which is ascribed to the favourable electron transfer ability of ZnO. In the meanwhile, holes gather on the T-PTCDI side, suggesting an electric field pointing from T-PTCDI to ZnO is obtained. Thus, due to the discrepancy of energy level and electron mobility between T-PTCDI and ZnO, an interfacial electric filed can be formed within the interface of these two components and can largely improve the electron transfer between them. At the same time, due to the π–π stacking interaction among the conjugated π system, H aggregation has been formed with the hybridization of T-PTCDI and ZnO. The π–π stacking interaction facilitates the transition and separation of electric charges, resulting in the formation of interfacial electric field and H aggregation in the T-PTCDI/ZnO composite which leads to the surface photovoltaic response increases exponentially. The effect of exterior electric field is usually applied to reflect the property of built-in field. Figure 4d shows the electric field-induced surface photovoltage spectrum of the T-PTCDI. No matter the photovoltaics response of T-PTCDI under electric field is positive or negative, it has no prominent variation compared with those without electric field. This phenomenon demonstrates that the variation of Y1 and Y2 resulting from the intrinsic π–π transition in conjugated system. Additionally, the built-in field of T-PTCDI is inert to exterior electric field due to its poor carrier mobility; thus, it is difficult for photo-generated charges to perform directional movement.

Figure 4e, f shows the FISPS of T-PTCDI/ZnO composite under positive and negative electric field, respectively. Since exterior electric field has slight influence on the built-in field formed by T-PTCDI itself, it would mainly affect the interfacial electric field formed between the T-PTCDI and ZnO. Obviously, the photovoltaics response increases drastically along with the enhancement of the positive electric field intensity, indicating the direction of the interfacial electric field is identical to the positive electric field, i.e., pointing from surface to inside.

On the micro-level, the direction of interfacial electric field points from T-PTCDI to ZnO, while T-PTCDI coats on the surface of ZnO on the macro-level. Therefore, the direction of the interfacial electric field points from skin layer to bulk phase, which is the same as the direction of the positive electric field. As shown in Fig. 4f, photovoltaics response can hardly be observed in the negative electric field-induced SPS, which means the direction of the negative electric field is opposite to that of the interfacial electric field. The exterior electric field suppresses the separation effect of the interfacial electric field to the photo-generated carrier, resulting in drastically decrease in the effect of charge’s separation and further poor photovoltaics response. The variation of photovoltaic response with the field conforms to the principle of field-induced surface photovoltage.

**Fig. 5:**
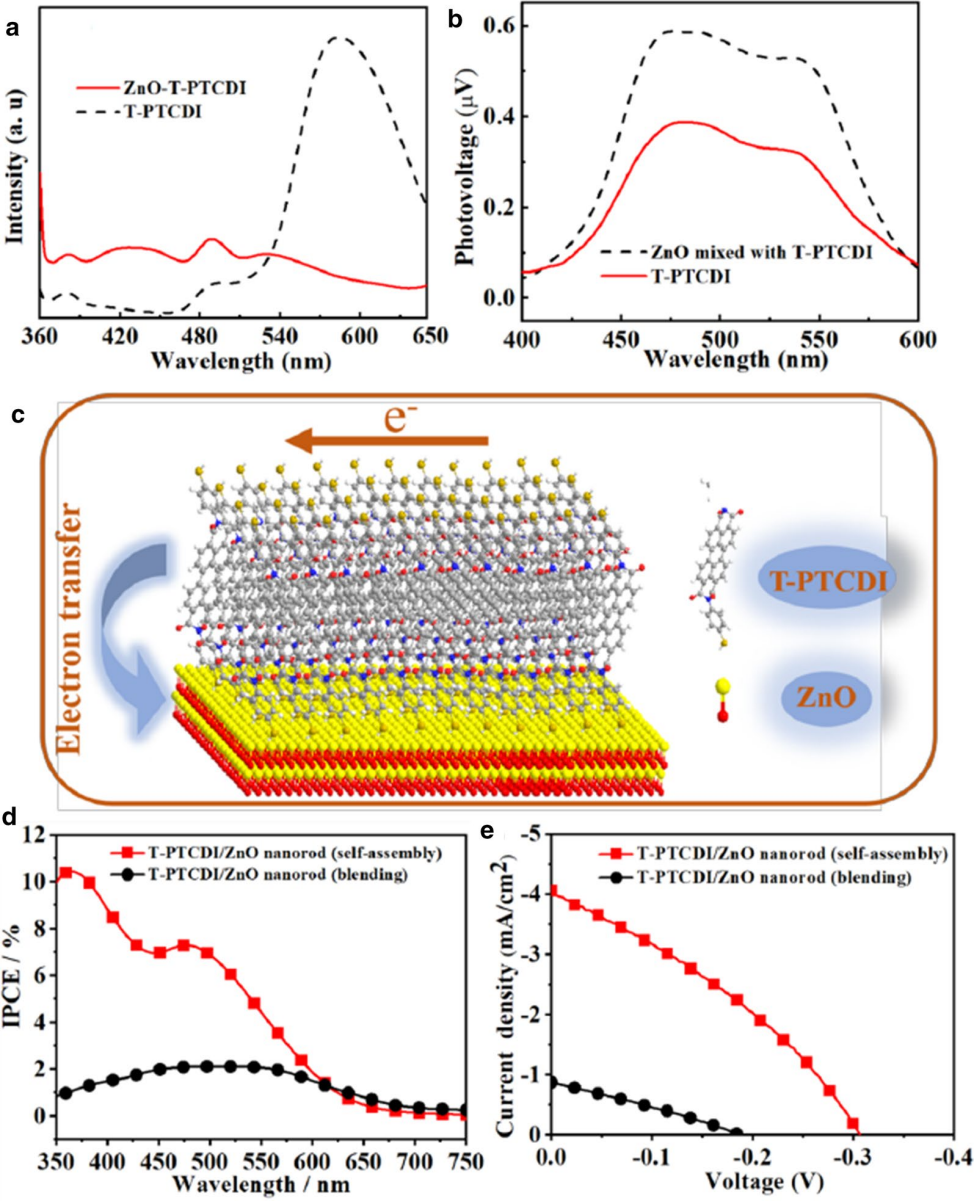
**a** Comparison of fluorescence spectra of T-PTCDI and ZnO-T-PTCDI (excitation wavelength of 325 nm) and **b** SPS spectrum of ZnO in ZnO/T-PTCDI blend system. **c** The sketch of molecules self-assembly and charge-transfer mechanism of T-PTCDI/ZnO nanorod composites. **d** Current–voltage characteristics and **e** IPCE spectra of T/PTCDI ZnO nanorod composite-based dye-sensitized solar cells under simulated solar illumination (AM 1.5G)

The charge transfer effect between ZnO and T-PTCDI can also be seen from fluorescence spectrum. As shown in Fig. 5a, the emission peak around 600 nm of T-PTCDI after compounding with ZnO almost can be hardly observed, indicating that the charge cannot be effectively recombined after isolation. This leads to fluorescence quenching.

Figure 5b shows the photovoltage response of T-PTCDI and the composite comprising of ZnO and T-PTCDI. It can be seen that the photovoltage response of the composite system has increased about twice which exhibits big difference in comparison with in-situ assembly system, indicating that there is poor interface assembly between ZnO and T-PTCDI. This demonstrates that good contact between ZnO and T-PTCDI is another key factor for photovoltage enhancement.

The mechanism of compounding and charge transfer of ZnO with T-PTCDI is shown in Fig. 5c. The increasing of the temperature results in the hydrolysis of hexamethylenetetramine which generates a large amount of OH^−^. Part of the alkali is consumed to generate ZnO and the rest part leads to the increase in alkalinity of the solution, resulting in the increased solubility of T-PTCDI under alkaline condition. Due to the coordination effect of Zn^2+^ with thiol functionality, self-assembly occurs in situ with ZnO during the growth stage. Benefit from the H-aggregates of T-PTCDI molecules and interface electric field between ZnO and T-PTCDI, electrons generated by T-PTCDI molecule can effectively transport in H-aggregates of T-PTCDI molecules and then transfer into ZnO nanorods, resulting in enhancement of surface photovoltage.

The monochromatic incident photo-to-electron conversion efficiency (IPCE), which was defined as the number of electrons generated by light in the outer circuit divided by the number of incident photons, is shown in the following Eq. (2):

2 $${\text{IPCE}}\left( \% \right) = \frac{{1240I_{{{\text{sc}}}} \left( {\upmu {\text{A}}\,{\text{cm}}^{ - 2} } \right)}}{{\lambda \left( {{\text{nm}}} \right)P_{{{\text{in}}}} \left( {{\text{W}}\,{\text{m}}^{ - 2} } \right)}}$$

where the constant 1240 is derived from unit conversion, the short-circuit photocurrent generated by monochromatic light is *I*_sc_, and *λ* is the wavelength of incident monochromatic light, *P*_in_ is the light intensity of which [16, 25, 26]. Figure 5d shows the incident monochromatic photon-to-current conversion efficiency (IPCE) curve for the solar cell prepared by T-PTCDI/ZnO nanorod composites. Compared with blended T-PTCDI/ZnO nanorod composites, the introduction of ordered self-assembly T-PTCDI/ZnO nanorod composites induces a significant improvement in IPCE throughout almost the whole wavelength region (350–650 nm), and from 2 to 7% in range of 450–500 nm. The overall power conversion efficiency of DSSC with the electrolyte containing self-assembly T-PTCDI/ZnO nanorod composites is about 0.4% (*J*_sc_ = 4.4 mA cm^2^, *V*_oc_ = 0.31 V, and ff = 0.32), which is larger than that of blended T-PTCDI/ZnO nanorod composites DSSC (0.05)% (*J*_sc_ = 0.86 mA cm^2^, *V*_oc_ = 0.19 V, and ff = 0.29) in Fig. 5e. This suggests that the enhancement in the solar cell performance upon the introduction of the T-PTCDI molecules ordered self-assembling on ZnO nanorods is due to the improved charge transfer efficiency, large light absorption range, scattering and enhancement in the electron lifetime [17, 27].

## Conclusion

In conclusion, in-situ ZnO nanocrystal growing method proposed in this work proves to be a powerful strategy for fabrication of molecular-level dispersed perylene bisimide/ZnO nanocrystals hybrids. The photovoltaics response of T-PTCDI hybridized with ZnO has significantly been enhanced compared to that of neat T-PTCDI, indicating that there exists a highly efficient charge transfer process between these two components. This process is driven by the interfacial electric field formed by the hybridization of T-PTCDI and ZnO as well as the formation of H-aggregates in the T-PTCDI. These lead to the effective improvement in electron mobility which further promotes transition and separation of the charges.

## Data Availability

All data are fully available without restriction.

## Abbreviations

T-PTCDI: Thiol-functionalized perylene-3,4,9,10-tetracarboxylic diimide
ZnO: Zinc oxide
LUMO: The lowest unoccupied molecular orbital
HUMO: The highest unoccupied molecular orbital
XRD: X-ray diffraction
TEM: Transmission electron microscopy
SAED: Selected-area electron diffraction
SPS: Surface photovoltaic spectra
FISPS: Field-induced surface photovoltage spectrum
IPCE: Incident photo-to-electron conversion efficiency
